# Overexpression and cytoplasmic localization of caspase-6 is associated with lamin A degradation in set of ovarian cancers

**DOI:** 10.1186/s40364-018-0144-9

**Published:** 2018-10-30

**Authors:** Callinice D Capo-chichi, Kathy Q Cai, Xiang-Xi Xu

**Affiliations:** 1 Institute of Applied Biomedical Sciences (ISBA), Unit of Biochemistry and Molecular Biology, Division of Molecular Biomarkers in Cancer and Nutrition, University of Abomey-Calavi, Abomey-Calavi, Benin; 2 Sylvester Cancer Center (SCCC), Ovarian Cancer Program, University of Miami, Miami, Florida USA; 3 Department of Pathology, Fox Chase Cancer Center, Philadelphia, PA USA

**Keywords:** Ovarian cancer, Active caspase-6, Cytoplasmic localization, Lamin A/C degradation, Immunofluorescence, Flow cytometry

## Abstract

**Background:**

In most women with ovarian cancer, the diagnosis occurs after dissemination of tumor cells beyond ovaries. Several molecular perturbations occur ahead of tumor initiation including loss of lamin A/C. Our hypothesis was that the loss of nuclear structural proteins A type lamins (lamin A/C) transcribed from LMNA gene and substrate for active caspase-6 maybe one of the molecular perturbations. Our objective is to investigate the association between the loss of lamin A/C and the overexpression of caspase-6 in ovarian cancer cells.

**Method:**

Western blotting and immunofluorescence were used to analyze the expression of lamin A/C and active caspase-6 in normal human ovarian surface epithelial (HOSE) cells, immortalized human ovarian surface epithelial cells and a set of seven ovarian cancer cell lines (including OVCAR3, OVCAR5, and A2780). The activity of caspase-6 was measured by densitometry, fluorescence and flow cytometry. Immunohistochemistry was used to evaluate the expression of caspase-6 in set of ovarian cancer tissues previously reported to have lost lamin A/C.

**Results:**

The results showed that HOSE cells expressed lamin A/C and no or low level of active caspase-6 while cancer cells highly expressed caspase-6 and no or low level of lamin A/C. The inhibition of caspase-6 activity in OVCAR3 cells increased lamin A but has no effect on lamin C; active caspase-6 was localized in the cytoplasm associated with the loss of lamin A.

**Conclusion:**

Overexpression and cytoplasmic localization of caspase-6 in ovarian cancer cells may be involved in lamin A degradation and deficiency observed in some ovarian cancer cells.

## Background

Ovarian cancer is the most lethal gynecological neoplasm and cause of death associated to cancer among women worldwide. Treatment for ovarian cancer is complex and the outcome after diagnosis is not satisfactory because the diagnosis occurs often after cancer cells had spread beyond the ovaries [1, 2]. It was reported that failure in ovarian cancer therapy occurs in 90% of cases [2]. It is becoming obvious that focusing on molecular abnormalities leading to cancer will help saving more women. Our former studies showed that lamin A/C expression was lost in ovarian cancer cell prior to nuclear deformation, chromosomal numerical instability, polyploidy and aneuploidy; all of which are hallmark for ovarian cancer [3, 4]. Lamin A was reported to be a substrate for caspase-6 [5–7]. As matter of fact, cleavage of lamin A/C was utilized as method to measure caspase-6 activity in whole cell assay [7]. Caspase-6 was reported to be activated by caspase-3 during apoptotic event [8–12]. To the best of our acknowledges, the link between cytoplasmic localization of activated caspase-6 and the loss of the nuclear structural protein lamin A in ovarian cancer was not yet reported. Our investigation demonstrated an inverse association between active caspase-6 and lamin A in ovarian cancer cell lines and tissues. We hypothesized that active caspase-6 may be involved in lamin A/C degradation leading to the loss of nuclear structural proteins A type lamins (lamin A/C) prior to nuclear anomalies leading to carcinogenesis.

## Methods

### Reagents

Tris-Base, glycine, sodium dodecyl sulfate, bis-acrylamide, nitrocellulose membrane, were purchased from Bio-Rad. Inc. (USA). NaCl, KCl, Tween-20, protease inhibitor PMSF, 2-mercaptoethanol, DTT, methanol, ethanol, EDTA, glycerol, sodium azide, sodium fluoride. The primary antibodies made in rabbit against lamin A/C, lamin A and cleaved lamin A were from Transduction Lab (USA). The primary rabbit antibodies for simultaneous detection of procaspase-6 and caspase-6 were from Sigma-Aldrich (USA) and Cell signaling. Peroxidase (HRP)-conjugated secondary antibody (anti-rabbit) made in goat was from Bio-Rad Inc. (USA). A Super Signal West Dura Extended Duration Substrate made by PIERCE was purchased from Thermo Scientific (Rockford, IL USA). Caspase-6 specific inhibitor drug A6339 (N-Acetyl-Val-Glu-Ile-Asp-aldehyde, Synonym: Ac-VEID-CHO) was purchased from Sigma-Aldrich, USA.

### Human ovarian surface epithelial and cancer cell cultures

- Human ovarian surface epithelial (HOSE) cells were established from ovaries obtained from prophylactic oophorectomies [13]. Specimen of fresh intact whole ovary was immersed in medium and send to the laboratory where the ovarian surface was gently scraped with a rubber policeman to collect cells. The ovarian tissues were then analyzed by pathologists to confirm the absence of microscopic tumors. HOSE cells were cultured in 105 + 199 media containing 15% FBS, streptomycin, and insulin.
- To prepare human “immortalized” ovarian (HIO) cells, HOSE cells were transfected with SV40 T-antigen and cultured in 105 + 199 (*V*/V) media containing 15% FBS, streptomycin, and insulin. HIO cells had a longer lifespan in culture and can be cultured up to 50 passages before undergoing senescence unlike HOSE cells that can only be maintained in culture up to 7 passages [3, 13].
- The OVCAR lines were previously established by Thomas Hamilton [3, 13] and the others (A1847, A2780, and ES2) were obtained from American Type Culture Collection. Ovarian epithelial cancer cell lines were cultured in DMEM with 10% FBS and streptomycin as previously reported [3].

### Western blot of ovarian primary surface epithelial, immortalized and cancer cells

Cells were cultured in six well dishes in respective media. Cells were washed with ice cold PBS and lysate with 200 μl RIPA buffer (Santa Cruz Biotechnology) in ice for 30 min. Aliquot of 2 μl was used for protein quantification with the Bio-Rad protein assay kit. Protein denaturation was achieved with 4× SDS buffer containing β-mercaptoethanol (2%) and glycerol (40%). Cell lysates were boiled for 5 min and stored at − 20 °C until needed for western blot analysis. Samples were loaded on 4–12% gradient SDS-polyacrylamide gels (Invitrogen) and run at 100 V for 2 h in tris-glycine buffer. Proteins were transferred from the gels to nitrocellulose membranes with transfer buffer containing tris-glycine and 20% methanol. The membranes were blocked with 5% milk in 1X TBS containing 0.1% Tween-20 (TBST) for 30 min at room temperature before incubation in primary antibody in 1% milk/TBST for 1 h at room temperature. The blots were washed 4 times for 10 min with TBST before incubation with HRP-conjugated secondary antibody in 1% milk/TBST for 1 h. The blots were washed 4 times for 15 min with TBST and the membranes were the incubated 3 min in Super Signal West Dura Extended Duration Substrate and exposed to x-ray film and a film developer for the detection of caspase-6 and lamin A/C. The same protocol was performed for the detection of ß-actin as loading control.

### Immunofluorescence microscopy

Cells were cultured on plastic slide up to 90% confluence in a six well plate. Cells were washed twice with PBS and fixed with 4% paraformaldehyde for 15 min. Fixed cells were washed three times with PBS, permeabilized with 0.5% Triton X-100 for 5 min and blocked with 3% BSA in PBS containing 0.1% Tween-20 for 30 min. For double staining of activated caspase-6 (p20) and lamin A, the slides were incubated for 1 h at 37 °C with primary antibody rabbit anti caspase-6 (p20) from Santa-Cruz biotechnology and with primary antibody mouse anti lamin A/C from Cell Signaling (USA). Subsequently slides were washed three times with 1% BSA in PBS and incubated in 1% BSA/PBS solution with secondary antibodies conjugated with AlexaFluor 488 (green fluorescence) and AlexaFluor 594 (red fluorescence) (Invitrogen, Carlsbad, CA). The nuclei were stained with DAPI (Invitrogen, Carlsbad, CA). Cells were mounted on glass slide and sealed in anti-fade reagent containing 0.1 M n-propyl gallate (pH 7.4) and 90% glycerol in PBS. A Zeiss microscope and AxioCam Camera and Axio Vision software 4.8 were used for image acquisition and processing [3].

### Flow cytometry analysis

The detection of caspase-6 activity was achieved using an APO LOGIX Carboxyfluorescein caspase-6 specific detection Kit. For the evaluation of caspase-6 activity, FAM-VEID-FMK Caspase-6 detection kit was used as recommended by the manufacturer. Cells were cultured in 96 well dishes in respective media for 24 h before the addition of Carboxyfluorescein (FAM) labeled peptide (VEID)-fluoromethyl Ketone (FMK) reagent for 2 h. FAM-VEID-FMK is a cell permeable probe that enters each cell and covalently binds to the reactive cysteine residue on the large subunit of the caspase-6 heterodimer (FAM- VEID-FMK-Caspase-6). The probe is sequestered and accumulated inside the cell while inhibiting further caspase-6 activity. Cells bounds to FAM-peptide-FMK can be detected by a flow cytometer, or a fluorescence microscope with the green fluorescent channel (488 nm). Cell sorting and flow analyses were performed on LSR Fortessa driven by BD’s FACS Diva software version 6.1.3 (Becton Dickinson, San Jose, CA).

### Caspase-6 mRNA suppression with siRNA

Caspase-6 down regulation was achieved with siRNA against caspase-6 was purchased from Santacruz biotechnology (USA). Cell were plated in 6 well dish 24 h prior to siRNA transfection with lipofectamine 2000 according to the manufacturer protocol (Invitrogen). Cells were washed twice with PBS and processed for immunoblotting as previously described [3, 13].

### Tumor specimen’s acquisition and immunohistochemistry

Tumor specimens processing and immunohistochemistry protocol were previously described [3]. In brief, our current study utilized archived tumor tissue microarrays and cell lines prepared from human ovaries obtained from prophylactic surgeries. Cancer tissues and cells lines were obtained from Fox Chase Cancer Center tumor bank, for research purpose. The use of the tumor tissues for research was approved by the Institutional Review Board (IRB) of both Fox Chase Cancer Center and of the University of Miami, Miller School of Medicine [3, 13]. The ovarian tumor tissue microarrays (duplicate core of 120 tumor tissues and 5 controls) and 20 prophylactic oophorectomies were provided by the Tumor Bank of Fox Chase Cancer Center as previously described in detail [3]. Immunostaining was performed using primary antibodies anti active caspase-6 made in rabbit along with the rabbit DAKO Envision TM+ System and the Peroxidase (DAB) Kit (Dako Carpinteria, CA) as previously described [3]. Negative controls were performed by replacing the primary antibodies with non-immunized IgG.

## Result and discussion

### Procaspase-6 and caspase-6 expression in ovarian normal, immortalized and cancer cells

**Fig. 1:**
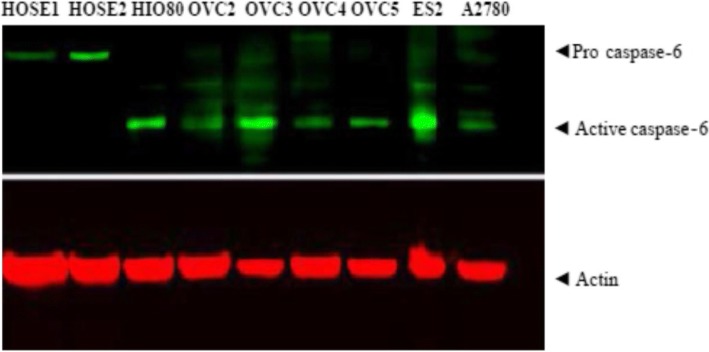
Immunofluorescence blot showing the expression of procaspase-6 and active caspase-6 in human primary surface epithelial cells (HOSE1 HOSE2), in human immortalized surface epithelial cells (HIO80) and in ovarian cancer cell lines OVCAR2, OVCAR3, OVCAR4, OVCAR5 and A2780. HOSE cells had constitutive procaspase-6 while proliferating HIO and cancer cells had constitutive active caspase-6

Licor-Odyssey immunofluorescence blot system was used to analyze Caspase-6 expressions in human primary surface epithelial cells (HOSE), in human immortalized surface epithelial cells (HIO80) and in ovarian cancer cell lines OVCAR5, OVCAR4, OVCAR2 and A2780 as shown in Fig. 1. After immunofluorescence revelation, OVCAR3 and ES2 had more activated caspase-6 (Fig. 1). The expressions of procaspase-6 was present in HOSE cells but not active caspase-6. In contrast, active caspase-6 was present in immortalized cells HIO80, in ovarian cancer cell lines OVCAR2–5, ES2 and A2780 but not in normal HOSE cells (Fig. 1). HOSE cells had constitutive procaspase-6 while proliferating HIO in culture and cancer cells had constitutive active caspase-6; actin was used as loading control (Fig. 1).

### Analysis of active or cleaved caspase-6 and cleaved Lamin A profile in ovarian normal, immortalized and cancer cells

**Fig. 2:**
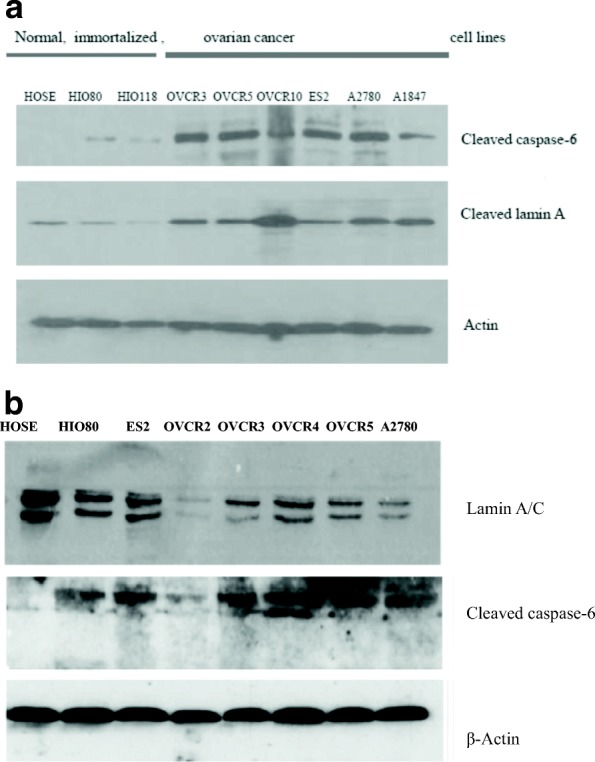
**a** Immuno-chemoluminescence blot showing the profile of cleaved lamin A/C and active caspase-6 in human primary surface epithelial cells (HOSE) in human immortalized surface epithelial cells (HIO80. HIO118) and in ovarian cancer cell lines OVCAR3, OVCAR4, OVCAR5, OVCAR10, ES2, A2780 and A1847. HOSE and proliferating HIO cells had no or weak cleaved lamin A/C and no or weak cleaved caspase-6 (active caspase-6). Ovarian cancer cells had high level of cleaved caspase-6 and cleaved lamin A/C. **b** Immuno-chemoluminescence blot showing the expression of lamin A/C and active caspase-6 in human primary surface epithelial cells (HOSE) in human immortalized surface epithelial cells (HIO80) and in ovarian cancer cell lines ES2, OVCAR2, OVCAR3, OVCAR4, OVCAR5 and A2780. HOSE cells had high level of lamin A/C but no active caspase-6 (cleaved caspase-6) while proliferating HIO and cancer cells had high level of active caspase-6 and weak expression of lamin A/C. Exceptionally OVCAR2 had weak lamin A/C and active caspase-6

The profile of cleaved lamin A and cleaved caspase-6 (active caspase-6) in normal HOSE cells, immortalized HIO80, HIO118, in ovarian cancer cell lines OVCAR3, OVCAR5, OVCAR10, ES2, A2780 and A1847 was shown on immuno-chemoluminescence blot (Fig. 2a). HOSE and HIO (passages < 10) cell lines had low or no active caspase-6 nor cleaved lamin A/C in contrast to most of ovarian cancer cell lines with high expression of active caspase-6 and cleaved lamin A (Fig. 2a). ES2 had less cleaved lamin A than the other cancer cells although its active caspase-6 level was higher (Fig. 2a).

### Analysis of active or cleaved caspase-6 and lamin A/C profile in ovarian normal, immortalized and cancer cells

Immuno-chemoluminescence blot showing the expression of lamin A/C, active caspase-6 and β-actin in human primary surface epithelial cells (HOSE) in human immortalized surface epithelial cells (HIO80) and in ovarian cancer cell lines ES2, OVCAR2, OVCAR3, OVCAR4, OVCAR5 and A2780, is shown in Fig. 2b. HOSE cells had high lamin A/C and no active caspase-6 (cleaved caspase-6) while proliferating HIO and cancer cells ES2 had active caspase-6 and weak expression of lamin A/C. Exceptionally for OVCAR2 with weak active caspase-6 and lamin A/C and active caspase-6 all other ovarian cancer cell lines had high expression of cleaved caspase-6 and weak expression of lamin A/C (Fig. 2b). The β-actin was used as loading control.

### Evaluation of active caspase-6 dynamic in normal and some ovarian cancer cells by immunofluorescence

**Fig. 3:**
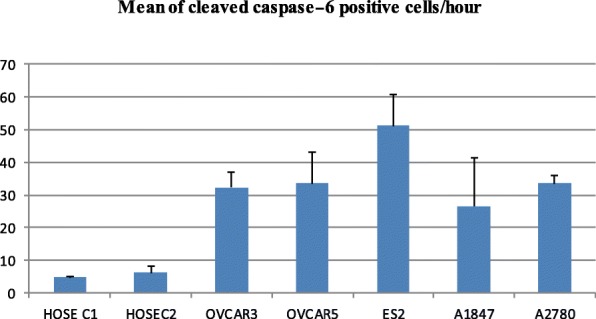
Immunofluorescence evaluation of active caspase-6 in cancer cells and control HOSE cells. All ovarian cancer cell lines expressed high level of active caspase-6 than control human ovarian surface epithelial cells (HOSE C1 and HOSE C2)

**Fig. 4:**
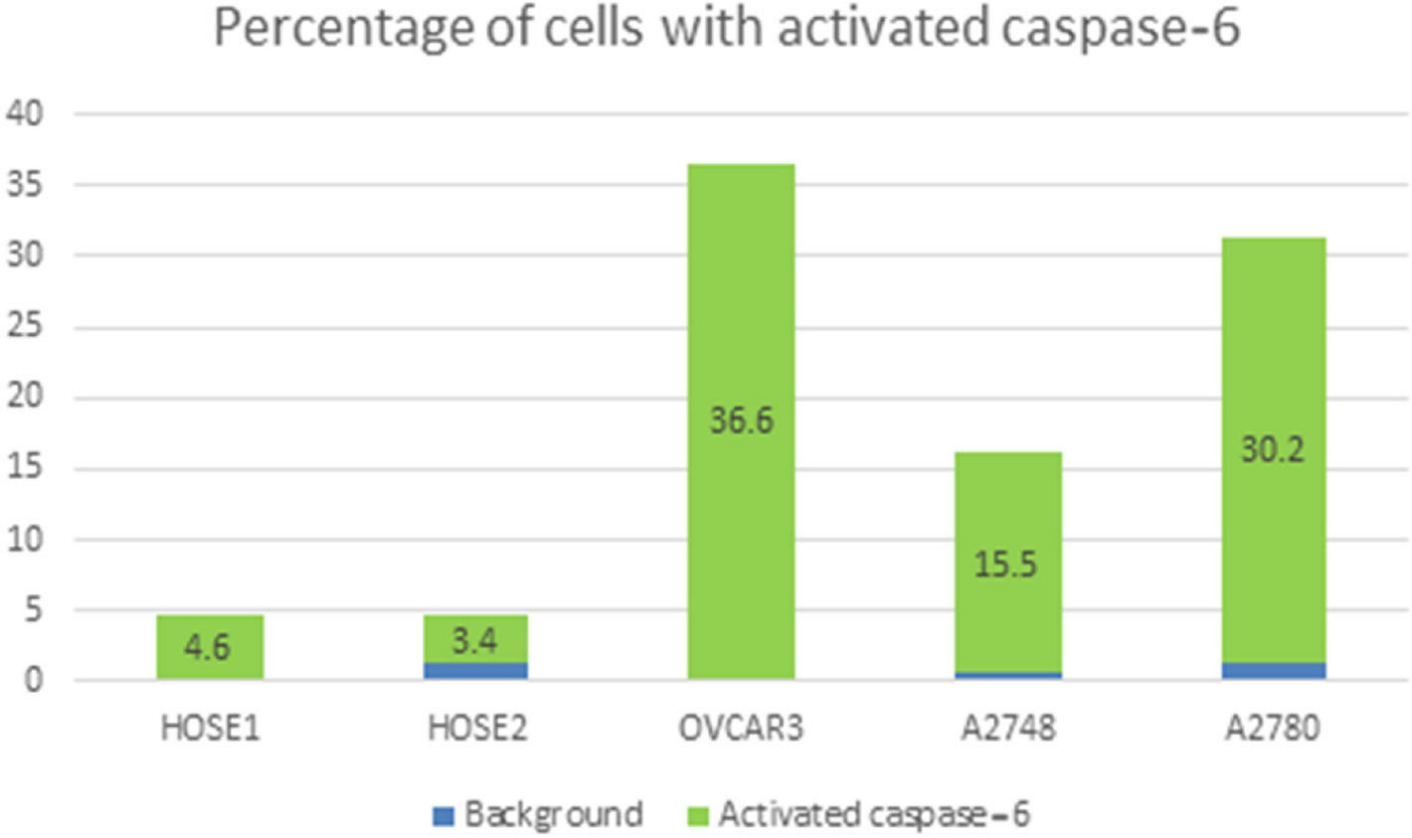
Histogram representing activated caspase-6 in primary human surface epithelial cells (HOSE) and in a set of cancer cell lines. The detection of caspase-6 activity was achieved using an APO LOGIX Carboxyfluorescein Caspase-6 Detection Kit. FAM-VEID-FM. Cell population with green fluorescence indicated cell entry of FAM-VEID-FMK covalently bound to active caspase-6 on cysteine residue among 10000 cells (events). Background fluorescence is indicated in blue histograms while activated caspase-6 is indicated by green histograms. Caspase-6 substrate measured by flow cytometry in HOSE cells control 1 (HOSE1), HOSE cells control 2 (HOSE2), OVCAR3, A2748 and A2780 are respectively 4.6%, 3.4%, 36.6%, 15.5% and 30.2%

The detection of caspase-6 activity was achieved using Carboxyfluorescein Caspase-6 Detection Kit, FAM-VEID-FMK caspase-6 (APO LOGIX). The assay was done according to the manufacturer protocol. Carboxyfluorescein (FAM)_labeled peptide fluoromethyl Ketone (FMK)-caspase-6 inhibitor [caspase-6 FAM-VEID-FMK Caspase-6 detection kit] is cell permeable compound that enter each cell and covalently binds to the reactive cysteine residue on the large subunit of the caspase heterodimer. The probe is sequestered and accumulate inside cell while inhibiting further caspase activity. Cells bounds to FAM-peptide-FMK can be detected by a fluorescence microscope (GFP Chanel) or a flow cytometer. For our experiment to detect active caspase-6 in cancer cells and HOSE controls, cells were plated in 12 well dish for 24 h and then incubated with FAM-VEID-FMK reagent for 2 h. Cells were fixed and cells with green fluorescence were counted among 100 cells. The results are shown in Fig. 3. All ovarian cancer cells displayed more cells with active caspase-6 than control HOSE C1 and HOSE C2. This experiment was repeated three times. The results are displayed in histogram (Fig. 3) as mean ± SD and are HOSEC1 5 ± 0.14; HOSEC2 6.2 ± 1.97; OVCAR3 32.2 ± 4.80; OVCAR5 33.56 ± 7.15; ES2 51.2 ± 9.61; A1847 26.55 ± 4.91 and A2780 33.6 ± 2.54. Flow cytometry also was used to evaluate caspase-6 activity in a set of HOSE and ovarian cancer cell lines. The results are displayed in Fig. 4.

### Evaluation of activated caspase-6 in normal and a set ovarian cancer cells by flow cytometer

The detection of caspase-6 activity was achieved using Carboxyfluorescein Caspase-6 Detection Kit, FAM-VEID-FMK Caspase-6 (APO LOGIX). The assay was done according to the manufacturer protocol. Cells were plated in 12 well dish for 24 h and then incubated with FAM-VEID-FMK reagent for 2 h. Green fluorescence emission of FAM-VEID-FMK covalently bound to the reactive cysteine residue on the large subunit of the caspase-6 heterodimer while inhibiting further caspase-6 activity, was detected by flow cytometry. Cell population with green fluorescence indicating cell entry of FAM-VEID-FMK covalently bound to active caspase-6 cysteine residue was counted in a population of 10000 cells (events). Flow analyses performed with two control human ovarian surface epithelial cells (HOSE1 and HOSE2) and three ovarian cancer cells lines (OVCAR3, A1847 and A2780) are shown in Fig. 4. Active caspase-6 is weak in HOSE1 and HOSE2 (4.6% and 3.4%) compared to cancer cell lines OVCAR3, A1847 and A2780 (36.6%, 15.5% and 30.2% respectively).

### Inhibition of caspase-6 activity and the effect on Lamin a/C expression in OVCAR3 cell line

**Fig. 5:**
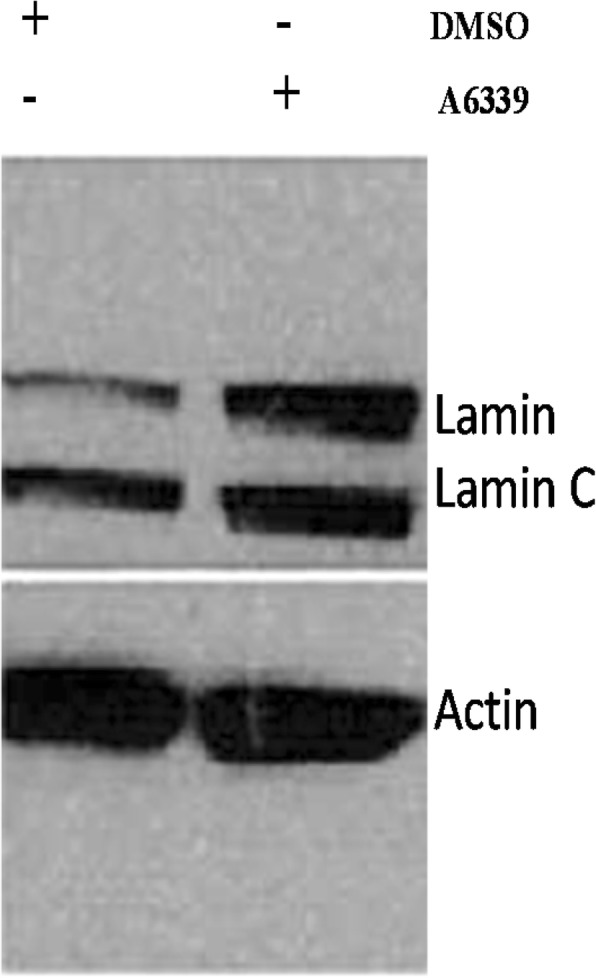
Western blot showing the Inhibition of caspase-6 activity by caspase-6 inhibitor reagent A6339 and the effect on lamin A/C expression. Inhibition of caspase-6 activity by A6339 increased lamin A/C expression in OVCAR3 cell line

We treated OVCAR3 cell line plated in 6 well culture dish with caspase-6 specific inhibitor drug A6339 for 24 h according to the manufacturer protocol. Cells were harvested and processed for immunoblotting with anti lamin A/C antibody as described above. The results showed an increase in lamin A/ C expression after inhibition of caspase-6 activity with drug A6339, actin was used as loading control (Fig. 5). This drug did not show restoration of lamin A/C in OVCAR5 cell line (data not shown).

### Immunofluorescence staining of active caspase-6 (p20) and lamin A in ovarian cancer cell line OVCAR3

**Fig. 6:**
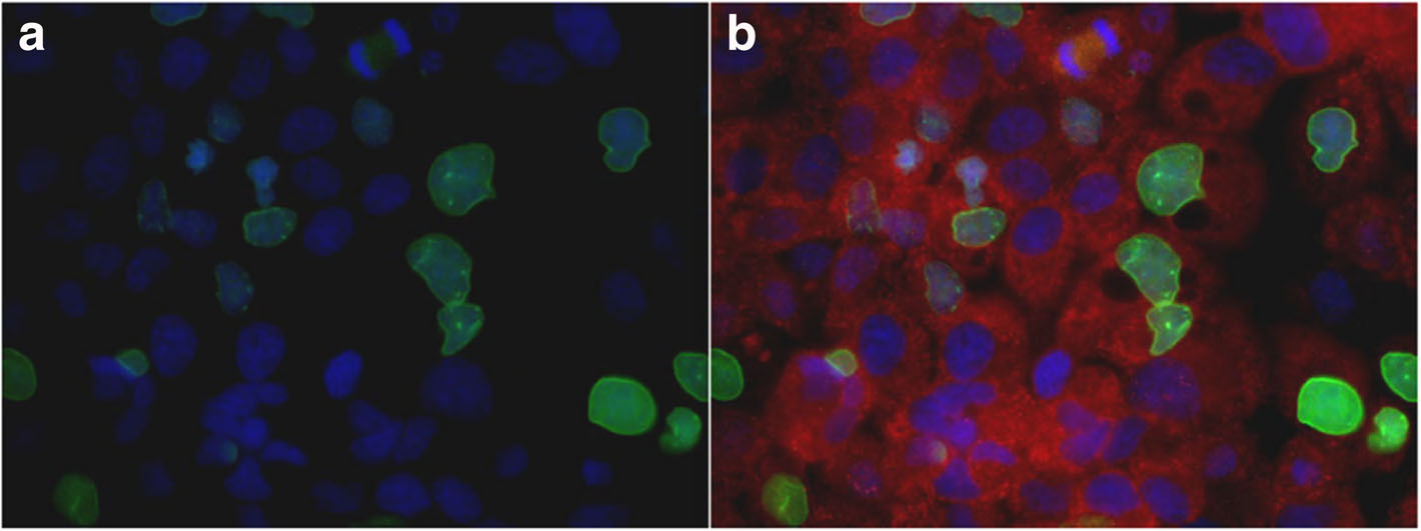
Immunofluorescence showing active caspase-6 (p20) and lamin A expression in OVCAR3 cell line. **a** solo image of OVCAR3 stained with lamin A/C (green) and Dapi (blue). **b** merged image of OVCAR3 stained with lamin A/C (green), active caspase-6 (p20) is in red and Dapi (blue). All OVCAR3 cells expressed activated caspase-6 p20 while most cells lost lamin A expression. Pictures were taken with 60× oil objective on ZEISS microscope

Double staining of active caspase-6 (p20) and lamin A specific antibodies was realized with OVCAR3 cell line and fluorescence microscopy images are displayed in Fig. 6. Image of lamin A immunofluorescence staining (green) along with nuclear counter stain with Dapi (blue) is displayed in Fig. 6a. Merged image of OVCAR3 stained with lamin A (green), caspase-6 (p20) in red and nuclear stain with Dapi (blue) is represented in Fig. 6b. All OVCAR3 cells expressed caspase-6 (p20) while most of cells had lost lamin A. Pictures were taken with 60× oil objective on ZEISS microscope.

### Immunofluorescence staining of caspase-6 (p20) and lamin a in ovarian cancer cell line OVCAR5

**Fig. 7:**
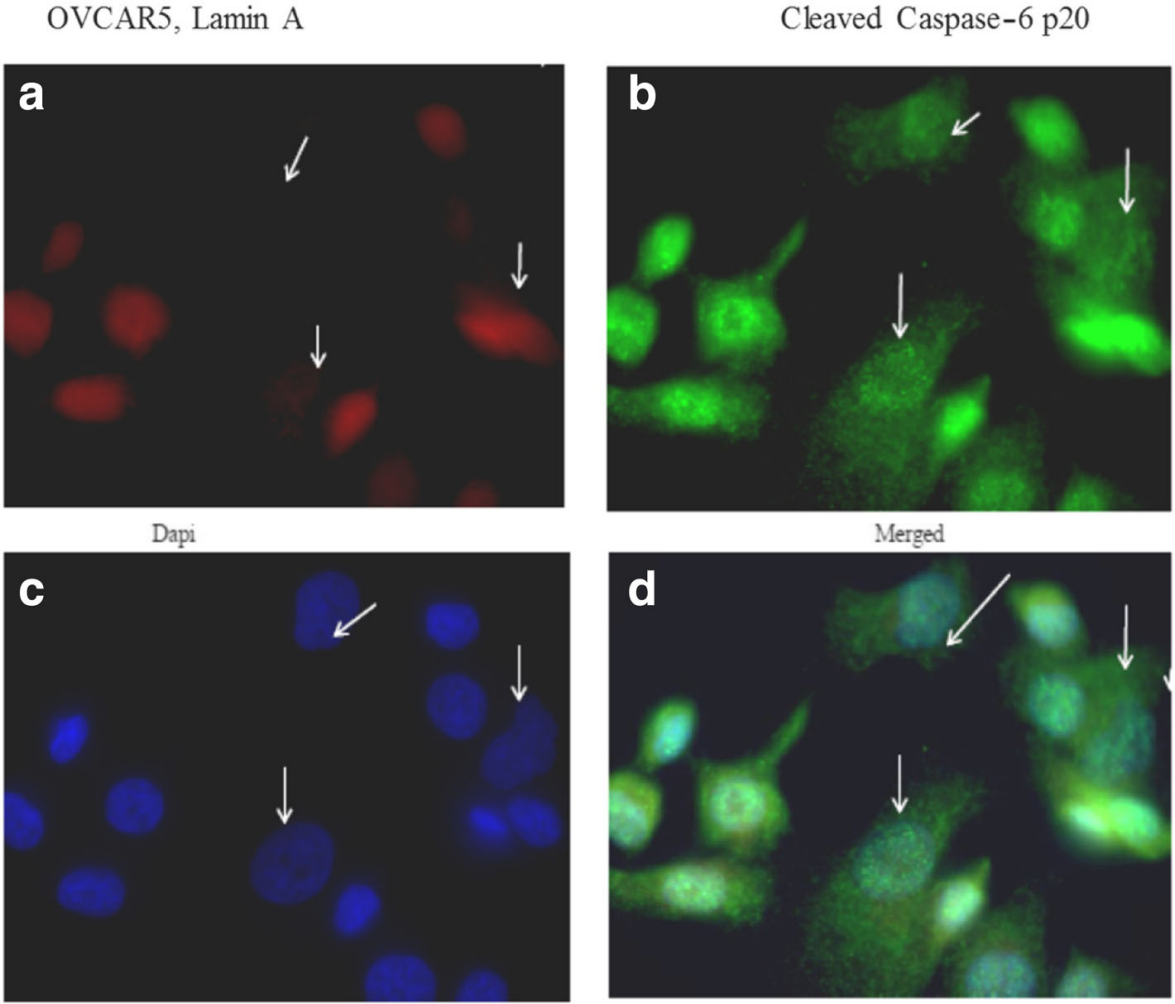
Immunofluorescence showing activated caspase-6 (p20) and lamin A expression in OVCAR5 cell line. (**a**) OVCAR5 stained with antibody against lamin A (red), and caspase-6 (p20) (**b**). Nuclear counter was stained with Dapi (**c**) and merged image of lamin A, caspase-6 (p20) and Dapi are shown in (**d**). The loss of lamin A is associated with the presence of caspase-6 in the cytoplasm as indicated by white arrows (**a**-**d**). Pictures were taken with 100× oil objective on ZEISS microscope

In OVCAR5 cell line, active caspase-6 (p20) and lamin A expressions were displayed in Fig. 7. Figure 7a represented OVCAR5 stained with lamin A (red); Fig. 7b represented OVCAR5 stained caspase-6 (green); Fig. 7c represented OVCAR5 stained with Dapi (blue) while Fig. 7d represented merged image of OVCAR5 stained with lamin A (red), caspase-6 (green) and Dapi (blue). All OVCAR5 cells expressed caspase-6 (p20) while most cells had lost lamin A. The loss of lamin A was associated with the presence of caspase-6 in the cytoplasm as indicated by white arrows (Fig. 7a-d). Pictures were taken with 100× oil objective on ZEISS microscope.

### Immunostaining of caspase-6 in ovarian epithelial transition to neoplasia and ovarian cancer tissues

**Fig. 8:**
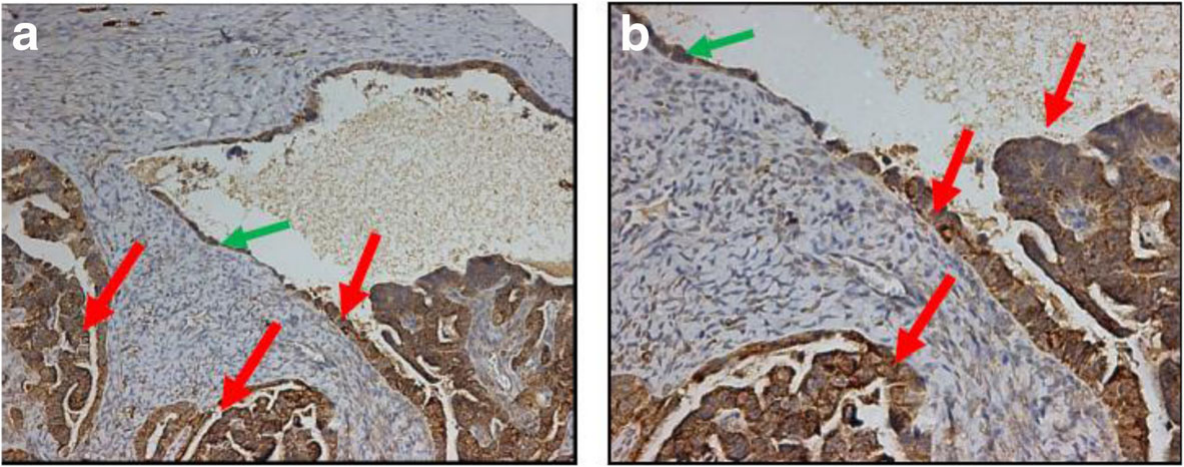
**a** Immunohistochemistry showing caspase-6 staining in ovarian epithelium transition to ovarian tumor. (**a**) Caspase-6 staining with 10× magnification; (**b**) Caspase-6 staining with 40× magnification. Activated caspase-6 is absent or weakly expressed in normal looking ovarian surface epithelial cells with normal nuclei (green arrow) while it is overexpressed in the cytoplasm of tumor cells with large nuclei (red arrow). Thus, active caspase-6 expression is absent in normal tissue (green arrows) and over expressed in the adjacent tumorous section

Active caspase-6 staining by immunohistochemistry in ovarian epithelium transition to ovarian neoplasia is displayed in Fig. 8; caspase-6 staining picture was taken with 10× (Fig. 8a) and 40× magnification (Fig. 8b). In normal epithelium active caspase-6 is absent or weak as indicated by green arrows (Fig. 8b). In contrast neoplastic, cells with enlarged nuclei displayed active caspase-6 in the cytoplasm (Fig. 8b). Thus, active caspase-6 expression is absent in normal tissue (green arrows) and over expressed in the adjacent tumorous section.

### Immunostaining of caspase-6 and lamin A in human ovarian surface epithelial, fallopian tube and ovarian cancer tissues

**Fig. 9:**
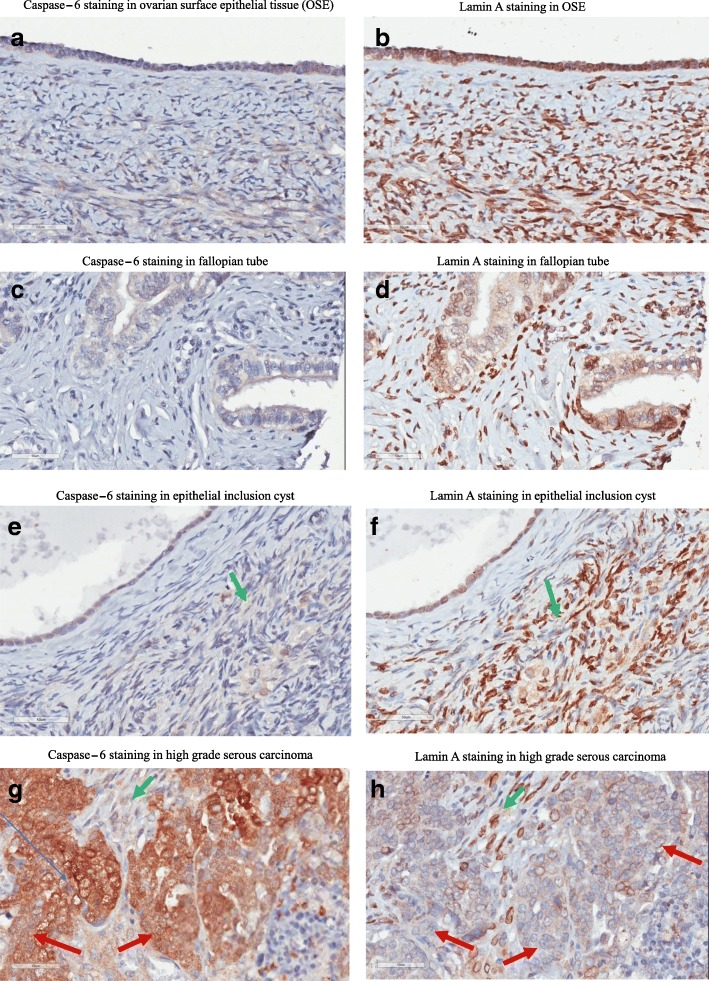
Immunohistochemistry with cleaved caspase-6 and lamin A antibody in human ovarian surface epithelial tissue (**a**, **b**), fallopian tube (**c**, **d**), epithelial inclusion cyst (**e**, **f**) and ovarian high grade serous carcinoma (**g**, **h**). No cleaved caspase-6 expression was observed in human ovarian surface epithelial and fallopian tube cells (**a**, **c**) in contrast lamin A expression was observed (**b**, **d**). Human epithelial inclusion cyst did not express cleaved caspase-6 but still expression lamin A (**e**, **f**). Human high grade serous carcinoma showed overexpression of cleaved caspase-6 in the cytoplasm (**g**) while the expression of lamin A is lost in the majority of cells (**h**) as indicated by red arrows. Seemingly normal epithelial cells with no cleaved caspase-6 expression still expressing lamin A as indicated by green arrows

Active caspase-6 and lamin A stainings are carried out in human ovarian surface epithelium, fallopian tube, epithelial inclusion cyst and high grade ovarian serous carcinoma and immunohistochemical images are displayed in Fig. 9. Immunohistochemistry with cleaved caspase-6 and lamin A antibodies on human ovarian surface epithelial tissue (Fig. 9a, b), fallopian tube (Fig. 9c, d), epithelial inclusion cyst (Fig. 9e, f) and ovarian high grade serous carcinoma (Fig. 9g, h). No cleaved caspase-6 expression was observed in human ovarian surface epithelial and fallopian tube cells (Fig. 9a, c) in contrast lamin A expression was observed (Fig. 9b, d). Human epithelial inclusion cyst did not express cleaved caspase-6 but still expressing lamin A (Fig. 9e, f). Human high grade serous carcinoma showed overexpression of cleaved caspase-6 in the cytoplasm (Fig. 9g) while the expression of lamin A is lost in the majority of cells (Fig. 9h) as indicated by red arrows. Seemingly normal epithelial cells with no cleaved caspase-6 expression still expressing lamin A as indicated by green arrows. caspase-6 is mostly expressed in the cytoplasm of cancer cells; pictures were taken with 40× magnification. Overall, ovarian cancer cells with abnormal nuclei had high level of active caspase-6 in the cytoplasm while normal looking ovarian epithelial cells with normal nuclei had low or no expression of active caspase-6.

### Immunostaining of caspase-3 and caspase-6 in human ovarian high grade serous carcinoma

**Fig. 10:**
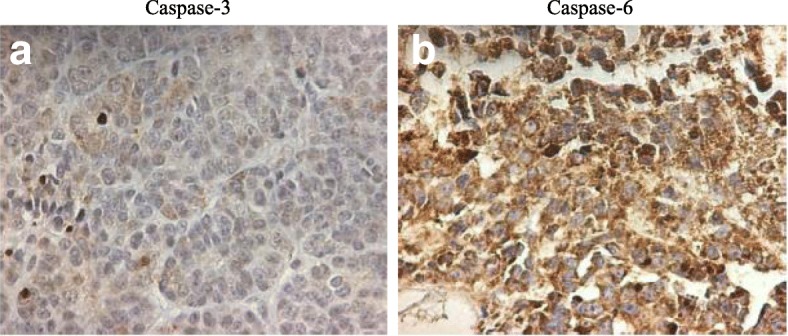
Immunostaining of caspase-6 and caspase-6 in human ovarian in high grade serous carcinoma. Caspase-3 is absent in ovarian carcinoma (**a**) while caspase-6 is heterogeneously expressed in the cytoplasm and in the nucleus in ovarian carcinoma (**b**). Down regulation of caspase-3 in ovarian carcinoma (**a**) is associated to the upregulation and cytoplasmic localization of caspase-6 (**b**)

Figure 10 displayed ovarian high grade serous carcinoma immunohistochemistry images of caspase-3 and cleaved caspase-6 staining. Caspase-3 is absent in ovarian carcinoma (a) while caspase-6 is heterogeneously expressed in the cytoplasm and in the nucleus in ovarian carcinoma (b). Down regulation of caspase-3 in ovarian carcinoma (a) is associated to the upregulation and cytoplasmic localization of caspase-6 (b).

### Caspase-3 is absent in OVCAR cell lines but can be restored with anti-cancer drug suberoyl bis-hydroxamis acid

**Fig. 11:**
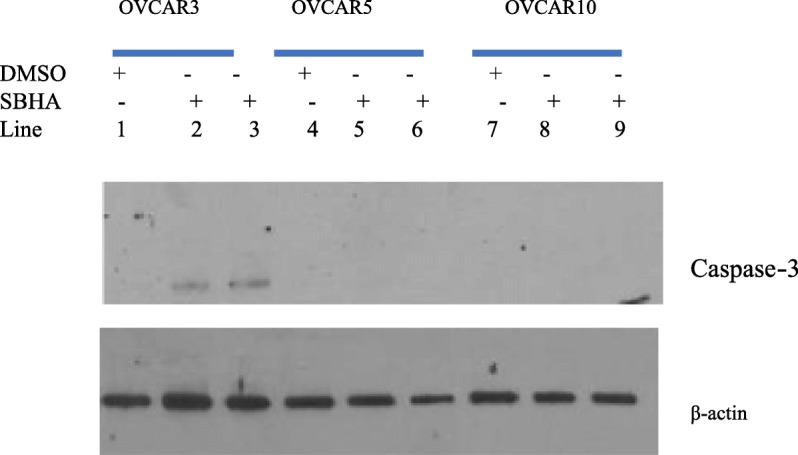
Western blot showed the absence of caspase-3 in ovarian cancer cell lines OVCAR3, OVCAR5 and OVCAR10. The anti-cancer drug suberoyl bis-hydroxamic acid (SBHA) was able to restore caspase-3 in OVCAR3 (line 2, 3) but not in OVCAR5 (lines 5, 6) and OVCAR10 (lines 8,9) cell lines. DMSO was used as mock treatment (lines 1, 4 and 7)

Western blot showed the absence of caspase-3 in ovarian cancer cell lines OVCAR3, OVCAR5 and OVCAR10 is displayed Fig. 11. Selected OVCAR cell line were treated to restore the apoptosis executioner caspase-3. The anti-cancer drug suberoyl bis-hydroxamic acid (SBHA) and a histone deacetylase inhibitor was able to restore caspase-3 in OVCAR3 (line 2, 3) but not in OVCAR5 (lines 5, 6) and OVCAR10 (lines 8, 9) cell lines. DMSO was used as mock treatment (lines 1, 4 and 7). Different mechanisms are involved in apoptotic failure of OVCAR cell lines.

### Suppression of caspase-6 with siRNA did not restored lamin A in OVCAR5 and A2780 cell lines

**Fig. 12:**
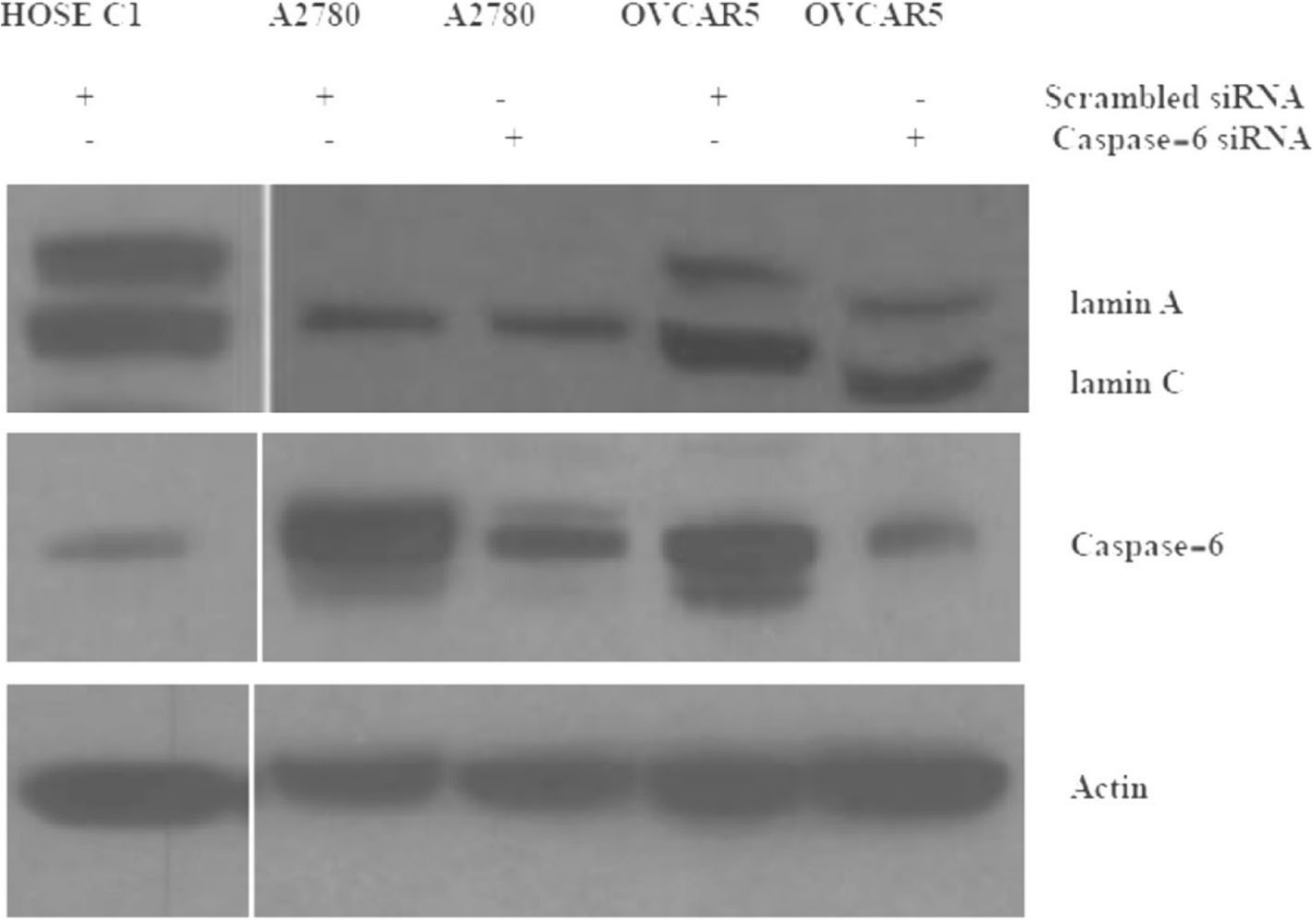
Immunoblot showing caspase-6 downregulation with siRNA. Primary human ovarian surface epithelial cells (HOSE), A2780 and OVCAR5 cell lines were transfected with siRNA for 72 h before cell lysates were collected for western blot analysis. HOSE cells displayed prominent levels of lamin A/C and low level of caspase-6 expression in contrast to cancer cell lines A2780 and OVCAR5. Caspase-6 downregulation was observed in A2780 and OVCAR5 cell lines but no effect was observed on lamin A/C expression

Primary human ovarian surface epithelial cells (HOSE) displayed prominent expression of lamin A/C and weak expression of active caspase-6 while ovarian cancer cells lines displayed prominent expression of active caspase-6 and low lamin A/C. The suppression of caspase-6 was achieved following transfection of siRNA in cancer cell lines OVCAR5 and A2780 for 72 h. The suppression of caspase-6 with siRNA did not restore the level of lamin A/C in OVCAR5 and A2780 cells; actin was used as loading control (Fig. 12).

## Conclusion

Lamin A/C expression is important for the maintenance and the integrity of nuclear envelope structure and nuclear morphology [3]. The destruction of lamin A/C in cancer including ovarian cancer is a complex molecular process which is not fully elucidated [3, 4]. From our study, we showed by immunoblotting, immunofluorescence, flow cytometry and immunohistochemistry that active caspase-6 was highly present in most ovarian cancer cell and tissues unlike normal ovarian epithelial cells or tissues. It was reported that lamin A showed proteolytic processing when incubated with recombinant active caspase-6 [14]. The nuclear localization of caspase-6 was commonly associated with the cleavage of nuclear lamin A and cell death (apoptosis) while cytoplasmic localization of active caspase-6 was not associated with instantaneous apoptosis [14, 15]. Our study showed that in ovarian cancer cell lines and in tumor tissues most of cleaved caspase-6 (active caspase-6) was localized to the cytoplasm while the expression of lamin A/C is absent. Our study also showed in some cancer cell lines the degradation of lamin A is related to active caspase-6 and can be abolished with caspase-6 inhibitor in OVCAR3 (Fig. 5) but not in OVCAR5 nor A2780 cell lines (data not shown). In these cell lines the downregulation of caspase-6 with siRNA did not restore lamin A/C either (Fig. 12) as observed for OVCAR2 cell line (Fig. 2b). Thus, the degradation of lamin A appears to be independent of caspase-6 in some ovarian cancer cell lines (A2780, OVCAR5 and OVCAR2) and suggested the existence of another route for lamin A degradation through phosphorylation by kinases perhaps [15]. Lamin A/C proteins are target for serine/threonine (SER/THR) kinases that are overexpressed in cancer cells [15]. These results will be displayed in a separate publication. Ovarian clear carcinoma ES2 cell line was the exception with low cleaved lamin A/C while active caspase-6 was high. Recent study reported that caspase-6 activation and lamin A degradation may dependent on high glucose metabolism [16]. Thus, ES2 cells may have failure in glucose uptake which may reduce lamin A degradation by caspase-6. It was shown that in normal cells, the apoptosis executioner caspase-3 cleaves caspase-6 which in turn cleaves lamin A/C prior to apoptosis ([5–11]). In our study, caspase-3 is absent while caspase-6 is overexpressed in ovarian carcinoma (Figs. 10 and 11). In cancer cells the concept of lamin A/C cleavage by caspase-6 prior to apoptosis is no longer applicable due to the absence of lamin A. Indeed, in cancer cells, there is an alteration of cell proliferation and apoptotic markers [12]. From our investigation active caspase-3 was absent in cells expressing cleaved caspase-6 as shown in some ovarian cancer cell lines or tumor tissues analyzed (Figs. 10 and 11); meaning that caspase-6 activity initiates caspase-3 activation in cell apoptosis is disrupted in cancer [9]. Treatment with anti-cancer drug suberoyl bis-hydroxamic acid (SBHA) can restore caspase-3 in OVCAR3 cell lines but not in OVCAR5 or OVCAR10. Thus, the mechanism leading to the loss of caspase-3 and overexpression of caspase-6 is different in each cell line and will be further investigated.

Overall, the overexpression and cytoplasmic localization of caspase-6 as well as degradation of lamin A/C in cancer cells seemed to be more associated to nuclear abnormalities initiating polyploidy, aneuploidy and chromosomal instability all of which are hallmarks for ovarian cancer [3, 4]. Further studies are needed to elucidate the switch from apoptotic pathways involving caspase-3, cleaved caspase-6 and lamin A/C degradation, to carcinogenesis pathways linking active caspase-6 and lamin A/C degradation. Thus, molecular events linking cleaved caspase-6 and lamin A/C degradation to apoptosis is uncoupled in most ovarian cancer cells probably due to the absence of caspase-3.

In most of ovarian cancer cell lines and tissues, the presence of active caspase-6 in the cytoplasm is associated with the loss of lamin A/C. This association suggested that an increased expression of caspase-6 and localization to cytoplasm may be involved in lamin A/C degradation prior to nuclear envelope structural defects, aneuploidy and chromosomal instability involved in tumor cell initiation. Beside caspase-6 activity, other molecular events (lamin A phosphorylation) may be involved in lamin A/C degradation and deficiency.

## Abbreviations

GFP: Green fluorescence protein
HIO: Human immortalized ovarian cells
HOSE: Human ovarian surface epithelial cells
HRP: Horseradish peroxidase
KCl: Potassium chloride
NaCl: Sodium chloride
NaF: Sodium fluoride
NaN3: Sodium azide
PBS: Phosphate buffered saline
PMSF: Phenyl-methyl-sulfonyl fluoride
SBHA: Suberoyl bis-hydroxamic acid
SDS: Sodium dodecyl sulfate
siRNA: Small interfering ribonucleotide acid
TBS: Tris buffer saline
TBST: Tris buffer saline plus Tween20
Tris-HCl: Tris-hydrochloride
